# Low recurrence rate of anchored conjunctival rotation flap technique in pterygium surgery

**DOI:** 10.1186/s12886-017-0587-z

**Published:** 2017-10-10

**Authors:** Dong Ju Kim, Jimmy K. Lee, Roy S. Chuck, Choul Yong Park

**Affiliations:** 1 Department of Ophthalmology, Dongguk University, Ilsan Hospital, 814, Siksadong, Ilsan-dong-gu, Goyang, Kyunggido 410-773 South Korea; 2 Department of Ophthalmology and Visual Sciences, Montefiore Medical Center, Albert Einstein College of Medicine, Bronx, NY USA

**Keywords:** Pterygium, Flap, Anchored, Recurrence, Nylon, Polyglactin

## Abstract

**Background:**

To report the recurrence rate for an anchored conjunctival rotation flap technique in primary pterygium surgery.

**Methods:**

Primary pterygium surgeries performed using anchored conjunctival rotation flap techniques (110 eyes in 110 patients) with a minimum follow-up of 12 months were reviewed. In this technique, a conjunctival flap is rotated to cover the bare sclera and suture-fixated with either 8–0 polyglactin (41 eyes) or 10–0 nylon (69 eyes). The recurrence rate was determined, and the two suture materials utilized were compared.

**Results:**

The recurrence rate was 2.71% (3 cases in 110 eyes) when an anchored conjunctival rotation flap technique was used and patients were monitored for 26.40 ± 17.09 months. Interestingly, the recurrences were only observed in polyglactin-sutured eyes. No recurrence was detected in nylon-sutured eyes. No other complications were observed in either group.

**Conclusions:**

The anchored conjunctival rotation flap technique for pterygium surgery has a relatively low recurrence rate. Nylon suture-fixation of the flap was found to be superior to polyglactin suture-fixation in preventing recurrence.

**Electronic supplementary material:**

The online version of this article (10.1186/s12886-017-0587-z) contains supplementary material, which is available to authorized users.

## Background

The use of conjunctival flaps is a well-established strategy to prevent recurrence after pterygium excision [1–4]. Covering the bare sclera with autologous conjunctival tissue has been shown to significantly reduce postoperative pain and inflammation [5].

The optimal surgical technique for primary pterygium should be safe, simple, and effective. Using anti-metabolites, such as mitomycin C, is a simple and effective method that can be used to decrease the recurrence rate [6, 7]. However, the long-term safety of this method remains unclear, and many serious delayed complications related to mitomycin C have been reported [7]. Radical surgeries with a purported recurrence rate near 0 %, such as “Pterygium Extended Removal Followed by Extended Conjunctival Transplant” (PERFECT), have recently been introduced [4, 8]. However, these radical procedures have not been popularized due to the extensive labor involved and the complexity of the technique [4, 8].

Previously, we proposed a modified conjunctival flap procedure called the “anchored conjunctival rotation flap” technique for use in primary pterygium surgery [9]. In this technique, a rectangular conjunctival flap, corresponding to the bare scleral area, is harvested from the superior conjunctiva. Then, the flap is rotated nasally around a limbal anchoring point and tightly sutured to the bare scleral area using polyglactin sutures. In our initial series of 35 cases (mean follow up of 32.26 months), we reported a recurrence rate of 8.6%, which was comparable to the recurrence rates observed with conjunctival autografts [9].

In this consecutive case-series study, we report our long-term experience with the anchored conjunctival rotation flap technique in primary pterygium surgery over 6 years. Additionally, we compared the recurrence rates using polyglactin and nylon sutures.

## Methods

### Subjects

This study followed the tenets of the Declaration of Helsinki and was approved by the institutional review board of Dongguk University, Ilsan Hospital, Goyang, South Korea. A retrospective review of the medical records of 185 consecutive primary pterygium surgeries using the anchored conjunctival rotation flap technique from September 2009 to March 2016 was conducted. The inclusion criteria were an age equal or under 80 years; no concurrent ocular surface pathology including severe dry eye and blepharitis; a minimum of 12 months of follow-up after the surgery, and no use of mitomycin C. A total of 128 eyes from 110 patients satisfied the minimum 12-month follow-up criterion. Fifty seven eyes failed to complete 12 month follow-up (179 eyes completed 6 month follow-up). To eliminate duplication bias when using two eyes from a single patient in the analysis, only the initially operated eyes were included in the final analysis (110 eyes from 110 patients).

### Clinical classification of pterygium

The clinical characteristics of the pterygium were classified using a modified classification system [10]. It considers the length of corneal involvement (stage), vascularity and tissue thickness (corneal and conjunctival part) of pterygium (Table 1).

**Table 1:** Clinical classification of pterygium

Stage (S)		
	1	corneal invasion less than 1 mm
	2	corneal invasion 1–2 mm
	3	corneal invasion 2–3 mm
	4	corneal invasion more than 3 mm
Vascularity (V)		
	1	minimal visible vessel equal to conjunctiva
	2	moderate vascularity more dense than conjunctiva
	3	severe vascularity with vessel congestion
Conjunctival tissue thickness (C)		
	1	flat tissue
	2	minimally elevated tissue
	3	tissue elevation up to 1 mm
	4	tissue elevation over 1 mm
Corneal tissue thickness (K)		
	1	flat tissue
	2	minimally elevated tissue
	3	tissue elevation up to 1 mm
	4	tissue elevation over 1 mm

### Intervention-anchored conjunctival rotation flap technique

All surgeries were performed by a single surgeon (C.P.) under local anesthesia at Dongguk University, Ilsan Hospital after obtaining informed consent from each patient. The resection margin of the primary pterygium was marked using Gentian violet, which included a 1-mm free margin up and down the pterygium neck and pterygium body and a 1-mm margin from the plica semilunaris. After ballooning the conjunctiva over the pteryigum using lidocaine mixed with epinephrine (lidocaine hydrochloride 1% and epinephrine 1:100,000), the blunt dissection of the conjunctiva was initiated at the pre-marked resection margin near the plica semilunaris. Then, conjunctival dissection was extended both superiorly and inferiorly to the limbal border/pterygium head. This procedure exposed the entire fibro-vascular stalk of the pterygium. Next, blunt dissection using a cotton-tip applicator further exposed the Tenon’s capsule around the pterygium while separating it from overlying conjunctiva. The pterygium head was meticulously separated from the underlying cornea using a No. 15 Bard-Parker blade. After the entire pterygium was freed from the ocular surface, the pterygium body and conjunctiva were excised *en bloc*. The remaining fibrovascular tissue over the medial rectus insertion was further removed about 2 mm beyond the conjunctival edges.

The detailed illustration of the procedure of the anchored conjunctival rotation flap was previously reported (Additional file 1: Video S1) [9]. Briefly, the superior nasal conjunctiva (approximately 6.0 × 8.0 mm) was bluntly dissected from the fornix to 1-mm from the limbus. Ballooning of conjunctiva and Tenon’s capsule facilitated the split thickness dissection of the flap. Residual layer of Tenon’s capsule was maintained at the donor site for prompt conjunctivalization. The flap was completed by cutting the limbal area and preserving the inferior limbal anchoring point (about 1 mm). The flap was rotated and sutured to the bare sclera using five (sometimes additional sutures were placed at the nasal flap margin) interrupted sutures. The flap usually covered 6 to 7 mm of the nasal conjunctiva from the limbus to plica semilunaris. The inferior flap edge would then face the limbus, while the superior flap edge faced the nasal fornix. The conjunctival tissue 1 mm within the limbus was cut to prevent exuberant corneal wound healing by the conjunctival epithelium.

The surgeon (C.P.) sutured the conjunctival rotation flap using 8–0 polyglactin 910 (Coated Vicryl, Ethicon) until October 2012 and then switched to 10–0 nylon (Ethilon, Ethicon) in November 2012. When using nylon sutures, the suture was cut approximately 3 mm distal from the knot for easy removal. A soft contact lens bandage (Acuvue Oasys, Johnson & Johnson) was applied for 1 week for every patient. Any remaining polyglactin suture material observed at the four-week visit was gently removed. All nylon sutures were removed at the one-week postoperative visit. The postoperative medication included 1% prednisolone acetate eye drops (four times a day) for 2 months, and 0.1% levofloxacin eye drops (four times a day) for 1 month. Regular postoperative ophthalmic examinations were conducted at 1 week, 1 month, 2 months, 6 months, 1 year, and every subsequent year.

### Outcome measures

Recurrence of pterygia was monitored on the basis of a operation site grading system as previously proposed [11]. Grade 1 to 3 were considered as “no-recurrence” and grade 4 was considered as “recurrence” (Table 2). Any complications related the surgery such as pyogenic granuloma, transient or permanent diplopia, restricted motility, and steroid induced glaucoma were monitored.

**Table 2:** The grading system of nasal conjunctiva after pterygium surgery

Grade 1	A normal appearance of the operated site
Grade 2	The presence of fine episcleral vessels in the excised area extending to the limbus but without any fibrous tissue
Grade 3	Fibrovascular tissue in the excided area reaching to the limbus but not invading the cornea
Grade 3	A true corneal recurrence, with fibrovascular tissue invading the cornea and across the limbus

### Statistical analyses

Statistical analyses were performed using SPSS Version 20.0 software (SPSS, Chicago, IL, USA). The chi-square test and Student’s t test were used to compare qualitative and continuous quantitative variables, respectively. A *p*-value <0.05 was considered significant.

## Results

A total of 110 eyes (age: 60.07 ± 8.58 years (mean ± standard deviation)) with 26.40 ± 17.09 months (mean ± standard deviation) follow-ups were included in this study.

The clinical characteristics of the studied eyes are described in Table 3. Polyglactin- and nylon-sutured eyes were not different in terms of the clinical grades of pterygia.

**Table 3:** Clinical characteristics of the pterygia studied

	Total (*n* = 110)	Groups by suture material		
		Polyglactin (*n* = 41)	Nylon (*n* = 69)	*p*-value
Sex (M:F)	53:57	21:20	32:37	0.384^a^
Age (years) (range)	60.07 ± 8.58 (39–80)	61.17 ± 8.11 (41–75)	59.38 ± 8.75 (39–80)	0.284^b^
Follow up (months) (range)	26.40 ± 17.09 (12–70)	39.98 ± 17.58 (12–70)	17.97 ± 9.90 (12–48)	<0.001^b^
Stage (grade) (range)	2.79 ± 0.67 (2–4)	2.93 ± 0.61 (2–4)	2.71 ± 0.69 (2–4)	0.097^b^
Vascularity (grade) (range)	2.56 ± 0.51 (2–3)	2.54 ± 0.50 (2–3)	2.58 ± 0.53 (2–3)	0.702^b^
Conjunctival thickness (grade) (range)	2.56 ± 0.53 (2–4)	2.61 ± 0.49 (2–4)	2.53 ± 0.56 (2–4)	0.445^b^
Corneal thickness (grade) (range)	2.59 ± 0.56 (2–4)	2.56 ± 0.55 (2–4)	2.61 ± 0.58 (2–4)	0.687^b^
Postoperative nasal conjunctiva status, n (%)				
Grade 1	101	36	65	
Grade 2	1 (0.9%)	1 (2.43%)	0 (0%)	
Grade 3	2 (1.81%)	1 (2.43%)	1 (1.44%)	
Grade 4	3 (2.72%)	3 (7.31%)	0 (0%)	0.049^a^

Data was described as mean ± standard deviation, *P* values were calculated using chi-square test^a^ and student’s t-test^b^

**Fig. 1:**
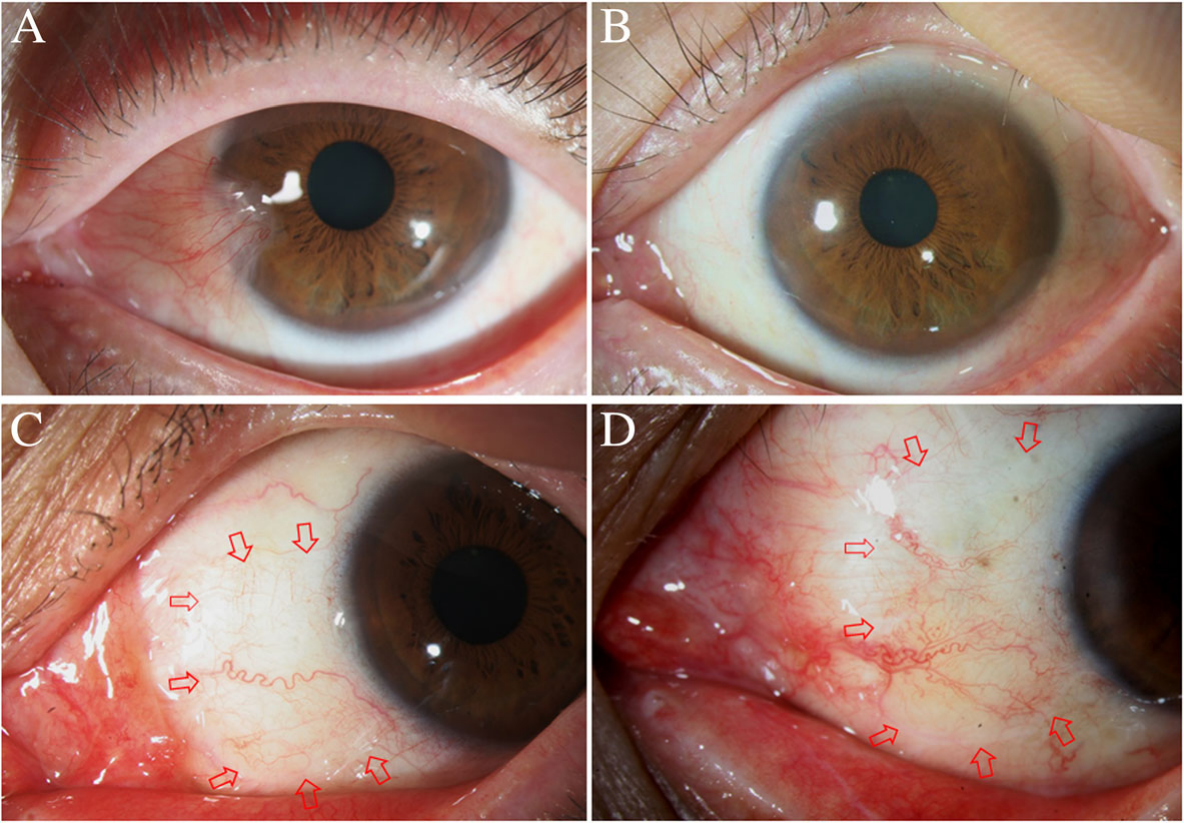
**a** & **b**: Primary pterygium before (**a**) and 48 months after (**b**) the anchored conjunctival rotation flap surgery in 54 year old male patient. Status of preoperative pterygium was graded as S_3_V_3_C_3_K_3_. The healing was accomplished with almost normal conjunctival appearance (grade 1). **c** & **d**: Representative pictures of the postoperative appearance of anchored conjunctival rotation flaps were shown. The conjunctival flaps were well maintained with normal vascularity. Arrows indicate the margin of the conjunctival flap. **c**: Forty eight months after the surgery in a 53 year old male patient (postoperative status grade 1). **d**: Twelve eight months after the surgery in a 68 year old male patient (postoperative status grade 1)

Anchored conjunctival rotation flap resulted in favorable postoperative result as shown in Fig. 1. The flaps were well maintained several years after the surgery (Fig. 1).

**Fig. 2:**
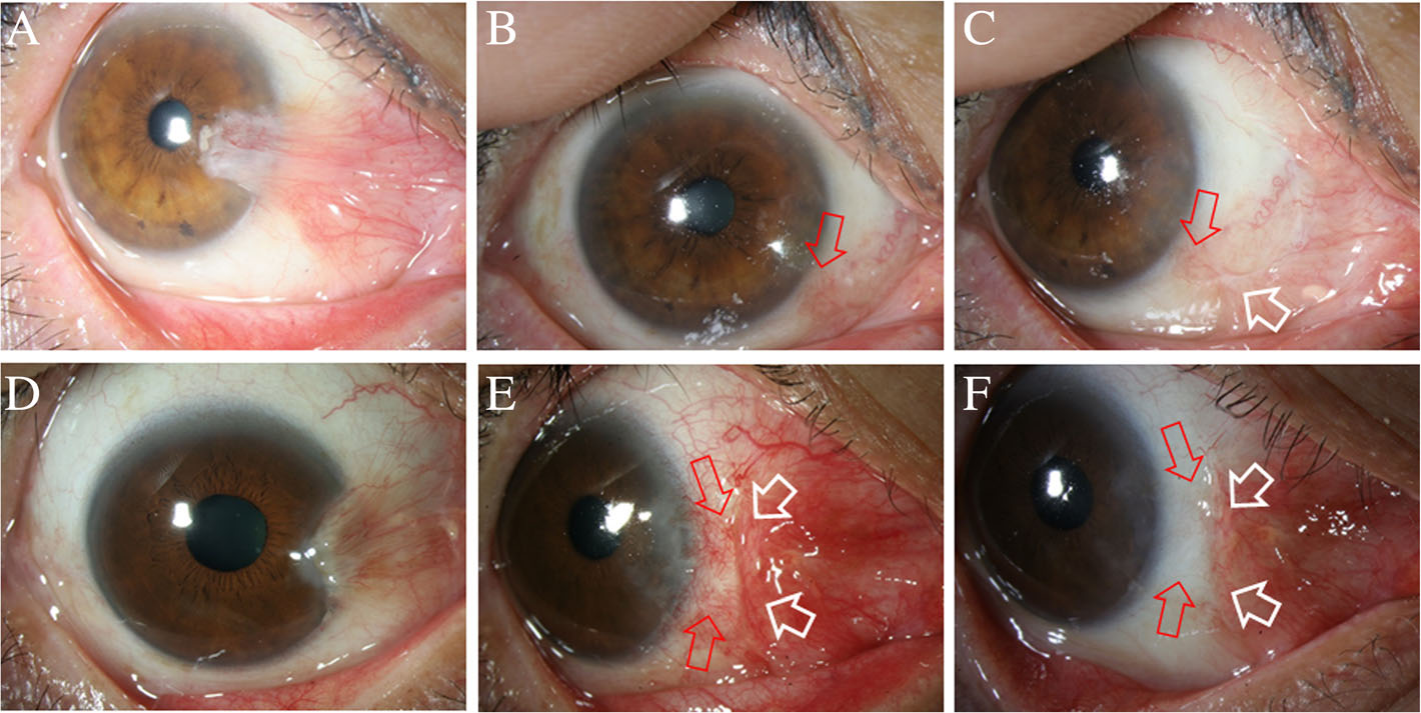
**a**: Thick and fleshy primary pterygium (S_4_V_3_C_4_K_3_) is observed with destruction of plica semilunaris and caruncle distortion in 61 year old female. **b** and **c**: Twelve month after surgery, focal presence of fine episcleral vessels (red arrow) reaching to the limbus is observed with thickened conjunctival wound edge (white arrow) (postoperative status, grade 2). **d**: Thick and atrophic primary pterygium (S_3_V_2_C_3_K_4_) is observed with dragging of plica semilunaris in 54 year old male. **e**: One month after surgery, significant contraction of conjunctival flap (red arrows) was observed with advancement of fibrovascular tissue (white arrows) from nasal conjunctiva. Monthly subconjunctival injection of bevacizumab (2.5 mg in 0.1 ml) and dexamethasone (0.5 mg in 0.1 ml) into the fibrovascular tissue was carried three times. **f**: Twelve month after surgery, fibrovascular tissue growth (white arrows) was halted with clear conjunctiva (red arrows) (postoperative status, grade 1)

Postoperative examination of the surgical site revealed three cases (2.72%) of recurrences (grade 4), two cases of grade 3 and one case of grade 2 (Fig. 2). All recurrences occurred with polyglactin-sutured eyes (7.31%) within 6 months postoperatively and this was statistically significant when compared to nylon sutured eyes (0%) (Table 3). Statistical analysis revealed significantly longer follow up for the polyglactin-sutured eyes (student’s t-test, *p* < 0.001). The recurrence rate in polyglactin-sutured eyes was significantly higher compared to nylon-sutured eyes (chi square test, *p* = 0.049). There were no recurrences among 18 second (opposite) operated eyes, which was monitored for a minimum of 12 months but not included in this study due to duplication bias.

In one case of a nylon-sutured eye, a significant contraction of conjunctival flap was noted with prominent fibrovascular tissue advancement from the nasal conjunctiva. Monthly subconjunctival injection of bevacizumab (2.5 mg in 0.1 ml) and dexamethasone (0.5 mg in 0.1 ml) into the fibrovascular tissue was administered three times to prevent recurrence (Fig. 2).

## Discussion

In this study, we observed a low recurrence rate (2.72%) for an anchored conjunctival rotation flap technique in primary pterygium surgery. Furthermore, no recurrence was observed during the follow-up period (mean of 17.97 months) when nylon sutures were used.

The recurrence rate after pterygium surgery is related to several factors, such as age, race, the clinical features of the pterygium, surgical experience, suture material, glue material, and the use of adjunctive agents, such as mitomycin C [12–15]. Because standardizing all parameters is implausible, a clear comparison of recurrence rates between studies are difficult. However, it is noteworthy that the recurrence rate for the anchored conjunctival rotation flap technique was relatively low compared to that of primary pterygium excision using conjunctival autograft (4.55~ 20%), which was recently reported for the same area and population [14, 16–18].

The key features of the anchored conjunctival rotation flap technique are as follows. First, with a wide resection of Tenon’s capsule beyond the border of the conjunctival resection margin (up to 2 mm in the nasal margin), the source of fibrovascular tissue for future recurrence can be reduced. Secondly, the partial preservation of the vascular network at the limbal anchoring area may play a role in enhancing flap viability and decreasing flap contraction. However, we admit that 1 mm vascular pedicle may not be sufficient to supply the entire conjunctival flap and total revascularization of flap bed is necessary for complete resolution of flap edema. Finally, another benefit of this technique is eliminating the need for a suture at the anchoring point and the easy recognition of flap orientation. For the aforementioned reasons, most surgeries utilizing this technique can be finished within 15 min.

There have been reports of polyglactin sutures inciting conjunctival reaction during the early postoperative period [19, 20]. Postoperative conjunctival inflammation and irritation induced by polyglactin sutures can lead to increased recurrence rates after pterygium excision. To date, there have been no studies comparing the pterygium recurrence rates with both types of sutures. In this study, we found that conjunctival rotation flaps sutured with nylon seemed to be associated with a lower recurrence rate (0% with nylon vs. 7.31% with polyglactin). Although the mean follow-up period was longer in polyglactin-sutured eyes, the statistical analysis reached clinical significance.

Recently, fibrin glue has been widely used to replace sutures for conjunctival flap surgery. Comparative studies investigating fibrin glue and suture techniques reported equal or lower recurrence rates after fibrin glue use [21–23]. Fibrin glue may increase flap vascularity in the early postoperative period and enhance flap survival as compared to sutures [24]. Fibrin glue anchoring of the conjunctival rotation flap is also feasible and is worth future investigation.

The large number of follow up loss (30.8%) is one of the major limitations of this study. This may increase the potential for a selection bias as those who failed to return may well not return to the surgeon who performed the failed procedure. However, it is noteworthy that 179 out of 185 eyes reviewed for this study completed their 6 months follow-up examination, although significant number of eyes failed to complete 12 month follow-up. There were no other recurrent cases in these 179 eyes except the reported 3 eyes. Another limitation of this study is its retrospective design. The use of two sutures (polyglactin and nylon) was not performed in the same period of time but sequentially. It is possible that there might be some minor changes in surgical technique which was unnoticed by the surgeon and the surgeon’s experience level might also affect the surgical outcome. Therefore, randomized prospective study might have enhanced the significance of our findings.

## Conclusions

We conclude that the anchored conjunctival rotation flap technique resulted in a low rate of recurrence after primary pterygium surgery. In addition, we recommend the use of a non-absorbable suture material, such as nylon, instead of an absorbable suture material, in order to further reduce the recurrence rate.

## Additional file

Additional file 1: Video S1. Surgical procedure of anchored conjunctival rotation flap surgery. (WMV 61807 kb)

## Abbreviations

C: Conjunctival tissue thickness
K: Corneal tissue thickness
S: Stage
V: Vascularity

## References

[1] Clearfield E, Muthappan V, Wang X, Kuo IC. Conjunctival autograft for pterygium. Cochrane Database Syst Rev. 2016;2:CD011349. 10.1002/14651858.CD011349.pub2 PMC5032146 26867004

[2] Al Fayez MF. Limbal-conjunctival vs conjunctival autograft transplant for recurrent pterygia: a prospective randomized controlled trial. JAMA Ophthalmol. 2013;131(1):11–6. 10.1001/archophthalmol.2012.2599 23307203

[3] Kenyon KR, Wagoner MD, Hettinger ME (1985). Conjunctival autograft transplantation for advanced and recurrent pterygium. Ophthalmology.

[4] Hirst LW. Recurrence and complications after 1000 surgeries using pterygium extended removal followed by extended conjunctival transplant. Ophthalmology. 2012;119(11):2205–10. 10.1016/j.ophtha.2012.06.021 22892149

[5] Kheirkhah A, Nazari R, Nikdel M, Ghassemi H, Hashemi H, Behrouz MJ. Postoperative conjunctival inflammation after pterygium surgery with amniotic membrane transplantation versus conjunctival autograft. Am J Ophthalmol. 2011;152(5):733–8. 10.1016/j.ajo.2011.04.013 21742306

[6] Koranyi G, Artzen D, Seregard S, Kopp ED. Intraoperative mitomycin C versus autologous conjunctival autograft in surgery of primary pterygium with four-year follow-up. Acta Ophthalmol. 2012;90(3):266–70. 10.1111/j.1755-3768.2010.01936.x 20528781

[7] Martins TG, Costa AL, Alves MR, Chammas R, Schor P. Mitomycin C in pterygium treatment. Int J Ophthalmol. 2016;9(3):465–8. 10.18240/ijo.2016.03.25 PMC4844053 27158622

[8] Cornelius CR. Recurrence rate and complications of Pterygium extended removal followed by extended Conjunctival transplant. Cornea. 2017;36:101-3. 10.1097/ICO.0000000000001026 27749451

[9] Kim SH, Oh JH, Do JR, Chuck RS, Park CY. A comparison of anchored conjunctival rotation flap and conjunctival autograft techniques in pterygium surgery. Cornea. 2013;32(12):1578–81. 10.1097/ICO.0b013e3182a73a48 24097183

[10] Park CY, Choi JS, Lee SJ, Hwang SW, Kim EJ, Chuck RS (2011). Cyclooxygenase-2-expressing macrophages in human pterygium co-express vascular endothelial growth factor. Mol Vis.

[11] Prabhasawat P, Barton K, Burkett G, Tseng SC (1997). Comparison of conjunctival autografts, amniotic membrane grafts, and primary closure for pterygium excision. Ophthalmology.

[12] Chui J, Di Girolamo N, Wakefield D, Coroneo MT (2008). The pathogenesis of pterygium: current concepts and their therapeutic implications. Ocul Surf.

[13] Tan DT, Chee SP, Dear KB, Lim AS (1997). Effect of pterygium morphology on pterygium recurrence in a controlled trial comparing conjunctival autografting with bare sclera excision. Arch Ophthalmol.

[14] Han SB, Jeon HS, Kim M, Lee SJ, Yang HK, Hwang JM, Kim KG, Hyon JY, Wee WR. Risk factors for recurrence after Pterygium surgery: an image analysis study. Cornea. 2016;35(8):1097–103. 10.1097/ICO.0000000000000853 27100658

[15] Zheng K, Cai J, Jhanji V, Chen H. Comparison of pterygium recurrence rates after limbal conjunctival autograft transplantation and other techniques: meta-analysis. Cornea. 2012;31(12):1422–7. 10.1097/ICO.0b013e31823cbecb 22643650

[16] Ha SW, Park JH, Shin IH, Kim HK. Clinical analysis of risk factors contributing to recurrence of pterygium after excision and graft surgery. Int J Ophthalmol. 2015;8(3):522–7. 10.3980/j.issn.2222-3959.2015.03.15 PMC4458656 26086001

[17] Kwon SH, Kim HK. Analysis of recurrence patterns following pterygium surgery with conjunctival autografts. Medicine (Baltimore). 2015;94(4):e518. 10.1097/MD.0000000000000518 PMC4602970 25634207

[18] Cha DM, Kim KH, Choi HJ, Kim MK, Wee WR. A comparative study of the effect of fibrin glue versus sutures on clinical outcome in patients undergoing pterygium excision and conjunctival autografts. Korean J Ophthalmol. 2012;26(6):407–13. 10.3341/kjo.2012.26.6.407 PMC3506813 23204794

[19] Wong VW, Rao SK, Lam DS (2007). Polyglactin sutures versus nylon sutures for suturing of conjunctival autograft in pterygium surgery: a randomized, controlled trial. Acta Ophthalmol Scand.

[20] Oguz H (2008). Polyglactin sutures versus nylon sutures for suturing of conjunctival autograft in pterygium surgery. Acta Ophthalmol.

[21] Pan HW, Zhong JX, Jing CX. Comparison of fibrin glue versus suture for conjunctival autografting in pterygium surgery: a meta-analysis. Ophthalmology. 2011;118(6):1049–54. 10.1016/j.ophtha.2010.10.033 21292327

[22] Ratnalingam V, Eu AL, Ng GL, Taharin R, John E. Fibrin adhesive is better than sutures in pterygium surgery. Cornea. 2010;29(5):485–9. 10.1097/ICO.0b013e3181c29696 20308876

[23] Sharma A, Raj H, Gupta A, Raina AV. Sutureless and glue-free versus sutures for Limbal Conjunctival autografting in primary Pterygium surgery: a prospective comparative study. J Clin Diagn Res. 2015;9(11):NC06–9. 10.7860/JCDR/2015/15689.6789 PMC4668443 26675383

[24] Kucukerdonmez C, Karalezli A, Zengin MO, Akova YA. Vascularization of conjunctival autografts in pterygium surgery: comparison of fibrin glue with sutures. Eur J Ophthalmol. 2014;24(6):824–9. 10.5301/ejo.5000466 24729145

